# Adamantyl Analogues of Paracetamol as Potent Analgesic Drugs via Inhibition of TRPA1

**DOI:** 10.1371/journal.pone.0113841

**Published:** 2014-12-01

**Authors:** Nieves Fresno, Ruth Pérez-Fernández, Carlos Goicoechea, Ibon Alkorta, Asia Fernández-Carvajal, Roberto de la Torre-Martínez, Susana Quirce, Antonio Ferrer-Montiel, M. Isabel Martín, Pilar Goya, José Elguero

**Affiliations:** 1 Instituto de Química Médica, IQM-CSIC, Madrid, Spain; 2 Departamento de Farmacología y Nutrición, Unidad Asociada de I+D+i al CSIC, Facultad de Ciencias de la Salud, Universidad Rey Juan Carlos, Alcorcón, Madrid, Spain; 3 Institute of Molecular and Cellular Biology, Universidad Miguel Hernández, Alicante, Spain; University of Hull, United Kingdom

## Abstract

Paracetamol also known as acetaminophen, is a widely used analgesic and antipyretic agent. We report the synthesis and biological evaluation of adamantyl analogues of paracetamol with important analgesic properties. The mechanism of nociception of compound **6a/b**, an analog of paracetamol, is not exerted through direct interaction with cannabinoid receptors, nor by inhibiting COX. It behaves as an interesting selective TRPA1 channel antagonist, which may be responsible for its analgesic properties, whereas it has no effect on the TRPM8 nor TRPV1 channels. The possibility of replacing a phenyl ring by an adamantyl ring opens new avenues in other fields of medicinal chemistry.

## Introduction

Although paracetamol (4-hydroxyacetanilide, acetyl-*p*-aminophenol, acetaminophen, APAP) [1]–[3] was found to be an effective analgesic more than a century ago, its mechanism of action is complex and the subject of continuous research mainly due to its extensive metabolism in animals and humans [4].

Several reports have described pathways of APAP to exert its analgesic activity. Unlike non-steroidal anti-inflammatory drugs (NSAIDs), whose analgesic and anti-inflammatory effects are related to their inhibition of the cyclooxygenase enzymes (COX-1 and COX-2), paracetamol is a weak anti-inflammatory agent with an absence of COX-related adverse effects [5]–[6]. In the brain and spinal cord paracetamol is metabolized by fatty amide hydrolase (FAAH) to *N*-arachidonoylphenolamine (AM404). AM404 is a known activator of the capsaicin receptor (TRPV1) [7] and the cannabinoid CB_1_ receptor system [8]–[9] both of which confer analgesia in the central nervous system. The main drawback found in the use of paracetamol is the mechanism-based inactivation (MBI) of cytochrome P450 enzymes (CYPs) [10]. APAP metabolites (e.g. N-acetyl-*p*-benzoquinoneimine (NAPQ1) have centered the attention because of their toxic actions causing hepatotoxicity [11]–[12]. Many articles have been devoted to this topic [13]–[17] and different attempts have been made to circumvent this problem. For instance, 3-hydroxyacetanilide is non hepatotoxic because the oxygen atoms of quinones can only be at 1,2-,*ortho*, or, 4-positions, *para* [18]. Other authors have designed new analgesics derived from the paracetamol metabolite (AM404) having an anandamide chain instead of the acetamido group [19]. The replacement of the methyl group by saccharin or an open form of it (SCP-1 and SCP-123) improves considerably its water solubility [20]. A proline prodrug was developed with this purpose [21] and a paracetamol analogue known as propacetamol is currently in the market. Note that paracetamol presents polymorphism [22]–[23]. Whereas researchers have been trying to avoid the formation of the metabolite responsible for APAP toxicity designing non-hepatotoxic paracetamol analogues [24], a new antinociceptive mechanism driven by activation of TRPA1 in the spinal cord by APAP metabolites such as NAPQ1 has been reported [25].

In August 2013, the FDA warned of rare but serious skin reaction associated with paracetamol. Despite the fact that it has been on the market for decades, paracetamol can be considered a “standalone” drug of which no effective analogues are known [26]. Some compounds which have been reported include the *N*-methyl derivative, and *N*-(1*H*-indazolo-5-yl), *N*-(4-hydroxy)acetamides and recently pyridinol-fused ring derivatives, but in all of them, the aromatic ring is present [27]–[29].

**Figure 1:**
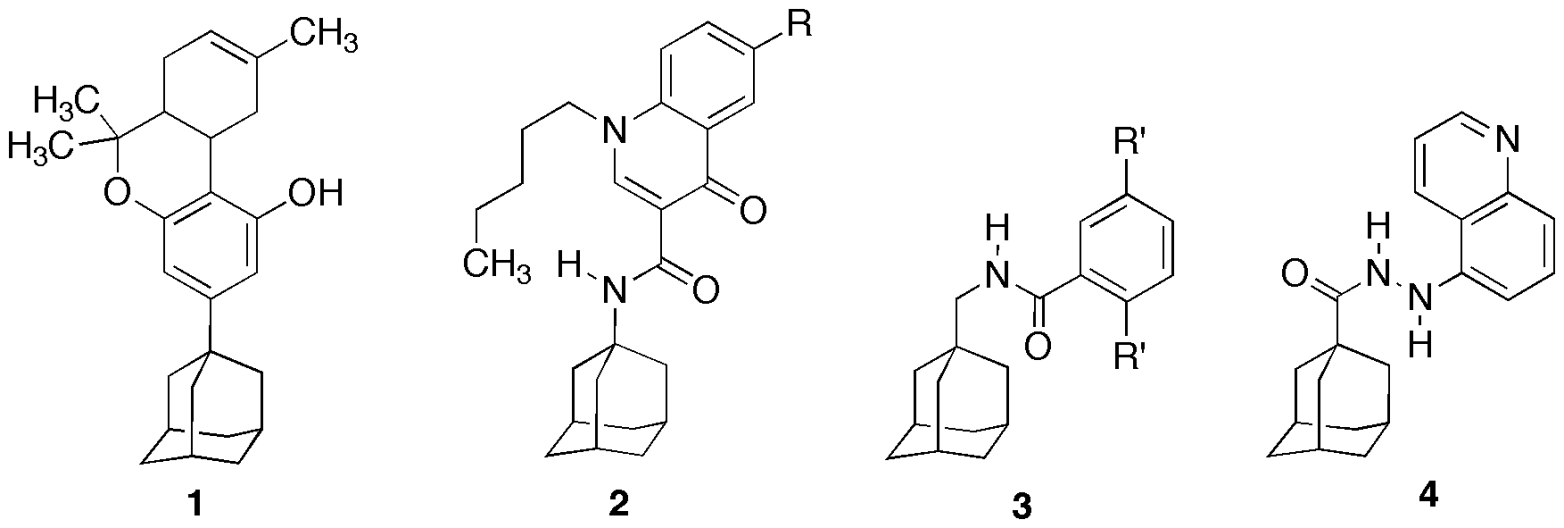
Some adamantyl derivatives with antiinflamatory and analgesic properties.

The approach proposed in the present paper is totally different being based on the hypothesis that the replacement of the phenyl ring of paracetamol by an adamantane ring with the same substituents in 1,4-positions, will yield analogues of paracetamol. Adamantyl derivatives are found in medicinal chemistry in very different domains [30]–[32]. Adamantane is one of the few singular hydrocarbon moieties that have been successfully employed in pharmaceutical industry. The most popular “hit” is amantadine used for the treatment of influenza and Parkinson's disease [33]. Related to analgesia and/or inflammation some analogues of Δ^8^-THC such as compound **1** have been reported (Fig. 1) [31]–[32]. Quinolone **2** binds selectively to the cannabinoid CB_2_ receptor being endowed with analgesic activity *in vivo* [34]. Some derivatives from **3** reported as P2X_7_ receptor antagonists acted as agents against rheumatoid arthritis [35]–[36]. Amongst the carbohydrazides, the most interesting is **4** developed by Abbott [37]. As can be seen, none of the structures represented in Fig. 1 is related to our compounds.

**Figure 2:**
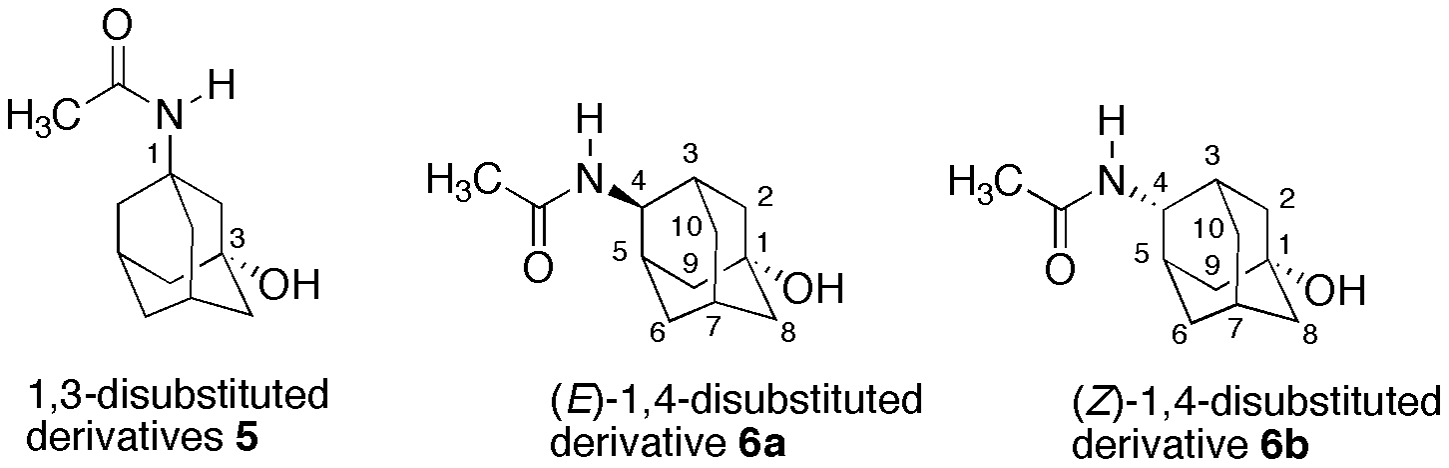
Adamantyl analogues of paracetamol.

In this work we report the synthesis and biological evaluation of compounds **5**, **6a** and **6b** (Fig. 2) in which the phenyl ring has been substituted by an adamantyl.

## Results and Discussion

### 1.1 Chemistry

**Figure 3:**
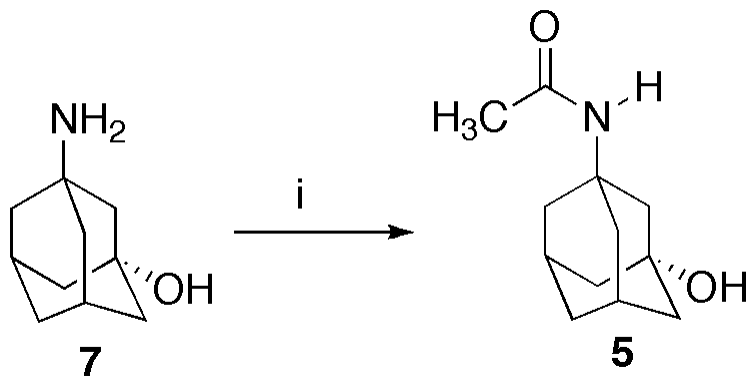
Synthesis of compound 5. Reagents and conditions: i) acetic anhydride, water, 0°C−rt.

The preparation of compound **5** is summarized in Fig. 3. Commercially available 3-amino-1-adamantanol (**7**) [38]–[39] was acetylated in water, the solvent of choice for green chemistry.

**Figure 4:**
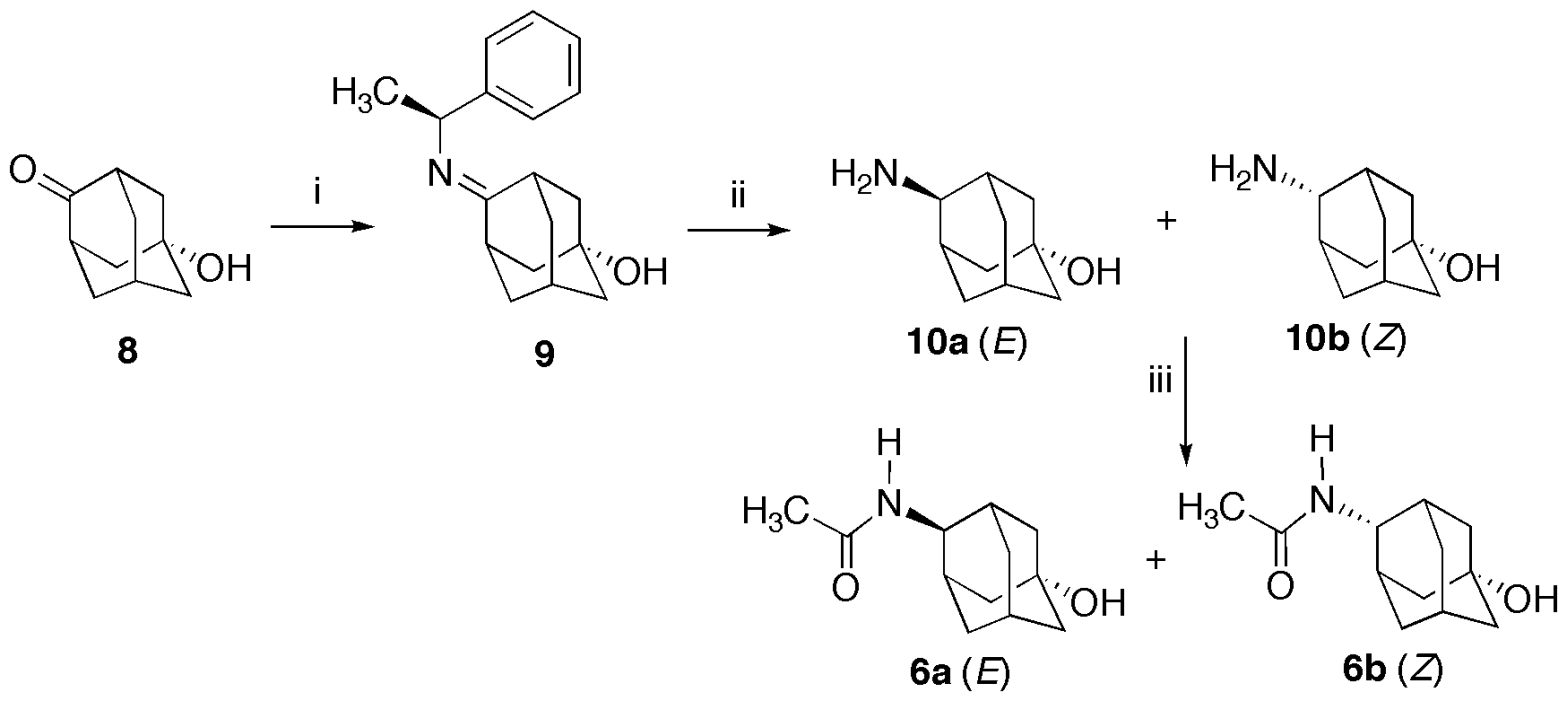
Synthesis of compounds 6a and 6b. Reagents and conditions: i) (i) *S*-α-methylbenzylamine, EtOH, reflux, 2 days; (ii) H_2_, 10% Pd-C, THF, rt; 12 h; (iii) acetic anhydride, water, 0°C−rt.

Compounds **6a** and **6b** were synthesized following the route summarized in Fig. 4 [40]. These compounds were obtained after a reductive amination and in three steps from the commercially available 5-hydroxy-2-adamantanone (**8**). The synthesis was carried out using the pure enantiomer of the *S*-α-methylbenzylamine and the 5-hydroxy-2-adamantanone refluxing in absolute ethanol. The intermediate compound **9** was hydrogenated. This hydrogenation with the catalyst Pd/C 10% could have been performed in two steps, isolating the benzylamino derivatives, but it was preferred an overnight hydrogenation to yield compounds **10a** and **10b**. The last synthetic step consisted of the acetylation in water of the mixture using the same conditions as for compound **5** yielding an *E/Z* mixture of compounds **6a** and **6b** in a 7∶3 ratio, with the E isomer being the major product. Compounds **6a** and **6b** were found in a paper by Gonzáles-Nuñez et al. [41] but with a different enriched mixture (*Z:E* 75/25) and not separated for fully characterization. In our case, compounds **6a** and **6b** were purified by automated semipreparative LC-MS for their characterization.

The ^13^C and ^1^H NMR spectroscopy in DMSO-*d_6_* of the mixture of **6a** and **6b** is interesting. The signals were assigned taking into account that **6a** was the major isomer (about 70%) and by means of 2D experiments. To check the assignments as well as the isomerism, we calculated the absolute shielding [42]-[43] (*σ*, ppm) at the GIAO/B3LYP/6-311+G(d,p) level and transform the absolute shielding into chemical shifts (*δ*, ppm, see Table S1).

### 1.2 Pharmacology

**Figure 5:**
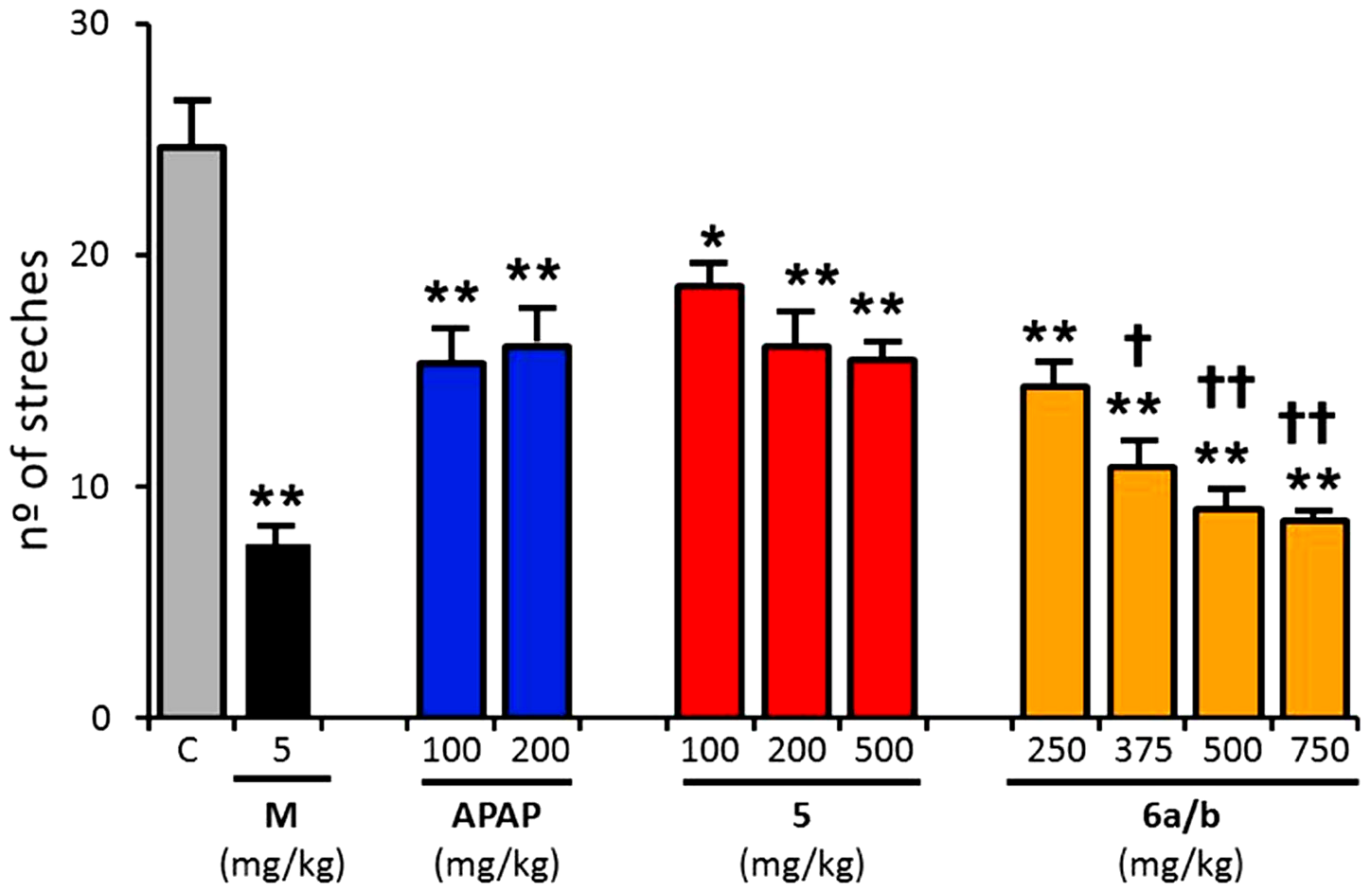
Analgesic activity of compounds 5 and 6a/b in comparison with paracetamol and morphine. Bars show the mean number of stretches induced by acetic acid (i.p., 2%) in mice treated with paracetamol (100, 200 mg/kg), morphine (5 mg/kg), compound **5** (100, 200 or 500 mg/kg), or compound **6a/b** (250, 375, 500 and 750 mg/kg). Data are given as mean ± SEM, with n = 15, and (* p<0.05, ** p<0.01 One way ANOVA to compare with control group.

The analgesic activity of compounds **5** and the **6a/b** mixture was compared to that of paracetamol and morphine in a model of visceral pain (Fig. 5). The intraperitoneal (i.p.) administration of acetic acid (2%) induced a typical writhing reaction, characterized by a wave of contraction of the abdominal musculature followed by extension of the hind limbs. After acetic acid administration, mice were placed in individual transparent containers and, after 5 min, the number of writhes was counted during a 10 min period. In naïve animals, the number of stretches induced by acetic acid was 25.4±0.8. Morphine (5 mg/kg, i.p.), an opioid accepted as the gold standard of analgesic drugs, notably decreased the number of writhes by 75%. Paracetamol (100 mg/kg), administered i.p. 30 minutes before acetic acid, was less effective than morphine reducing only by 40% the nociceptic behavior. Furthermore, a 2-fold increment in the paracetamol dose did not result in an increase of its analgesic effect in this pain model (Fig. 5).

Akin to the reference analgesic agents, compounds **5** and **6a/b** were administered i.p. 30 minutes before the injection of acetic acid (Fig. 5). The solubility of these derivatives was significantly higher than paracetamol allowing their testing at higher doses, which is a desirable property. Both compounds (**5** and **6a/b**) were able to induce a significant antinociceptive effect that was more potent for compound **6a/b**. As illustrated in Fig. 5, compound **5** displays a similar 40% antinociceptive activity to that recorded with paracetamol. This analgesic effect of compound **5** displayed some dose-dependency and saturated at 200 mg/kg. In contrast, compound **6a/b** exhibited stronger analgesic activity than compound **5**, and a clear dose-dependent analgesic activity that saturated at 750 mg/kg. At saturation, compound **6a/b** displays an analgesic efficacy significantly higher than paracetamol and similar to morphine. Taken together, these results suggest that compound **6a/b** is a more potent analgesic than paracetamol.

We next investigated the mechanism of action underlying the analgesic activity of analogue **6a/b**. First, we evaluated a possible action on cannabinoid receptors. For this purpose, compound **6a/b** was administered 30 minutes before the selective CB1 AM251 (3 mg/kg i.p.) and CB2 AM630 (1 mg/kg, i.p) antagonists, and the writhing test was carried out 30 minutes after the administration of the antagonists. Neither the CB1 nor the CB2 antagonist was able to block the antinociceptive activity of compound **6a/b** (number of stretches 13±1 (AM251) and 15.5±1.7 (AM630). Thus, the cannabinoid system does not appear to play a significant role in the antinociception induced by the paracetamol derivatives **5** and **6a/b**.

An alternative mechanism that we evaluated was the potential inhibition of cyclooxygenase enzymes COX-1 or COX-2. Our findings indicate that, while paracetamol inhibited COX-2 with an IC_50_ of 7.08±1.62 mM, compound **6a/b** did not affect the enzymatic activity at 10 mM. This result implies that inhibition of COX enzymes does not underlie the *in vivo* analgesic activity of compound **6a/b**.

**Figure 6:**
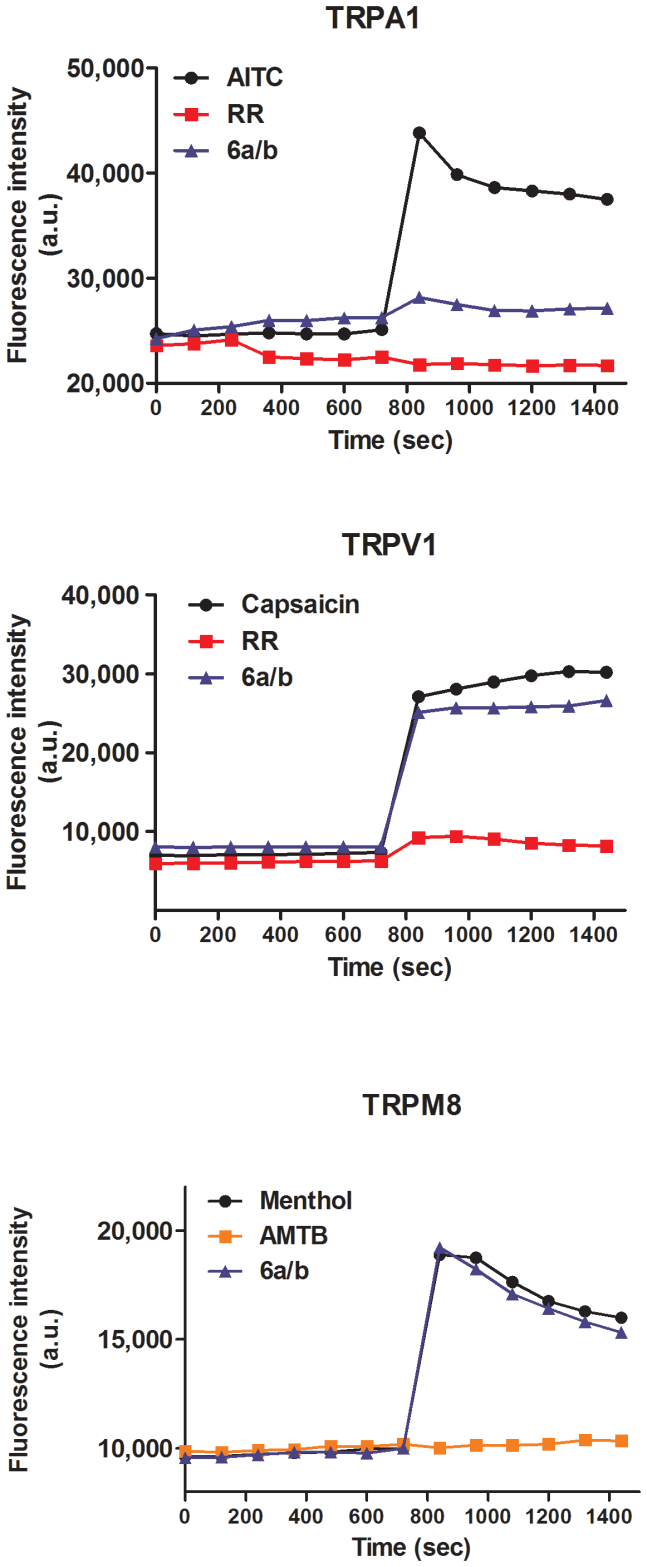
Functional expression of the TRP channels in the cell lines. Fluorescence time course for cells treated with TRP antagonists (squares) or agonist (circle). TRP-mediated Ca^2+^ influx was observed upon addition of agonist in the seven cycle, while the addition of the antagonist (squares) antagonized this response. The effect of the compound **6a/b** at 50 µM is shown (triangles). TRPA1 was activated with 100 µM AITC, TRPV1 was activated by 10 µM capsaicin and TRPM8 was activated with 100 µM menthol. Ruthenium red (RR) was at 10 µM, and AMTB was used at 100 µM.

Because of the pivotal role of thermoTRP channels in pain transduction [44]–[46], we hypothesized that the analgesic activity of compound **6a/b** may be due to a direct inhibition of some these channels. Among the thermoTRPs, TRPM8 (“melastatin”), TRPV1 (“vanilloid”) and TRPA1 (“ankyrin”) are the most validated in pain signaling. TRPV1 is considered a molecular integrator of noxious heat stimuli in nociceptors [47], TRPM8 is a pivotal sensor for cold stimuli, and TRPA1 is a unique sensor of noxious environmental stimuli [45]–[48]. Therefore, we selected these channels to evaluate if any of them was the target of compound **6a/b** (Fig. 6).

**Figure 7:**
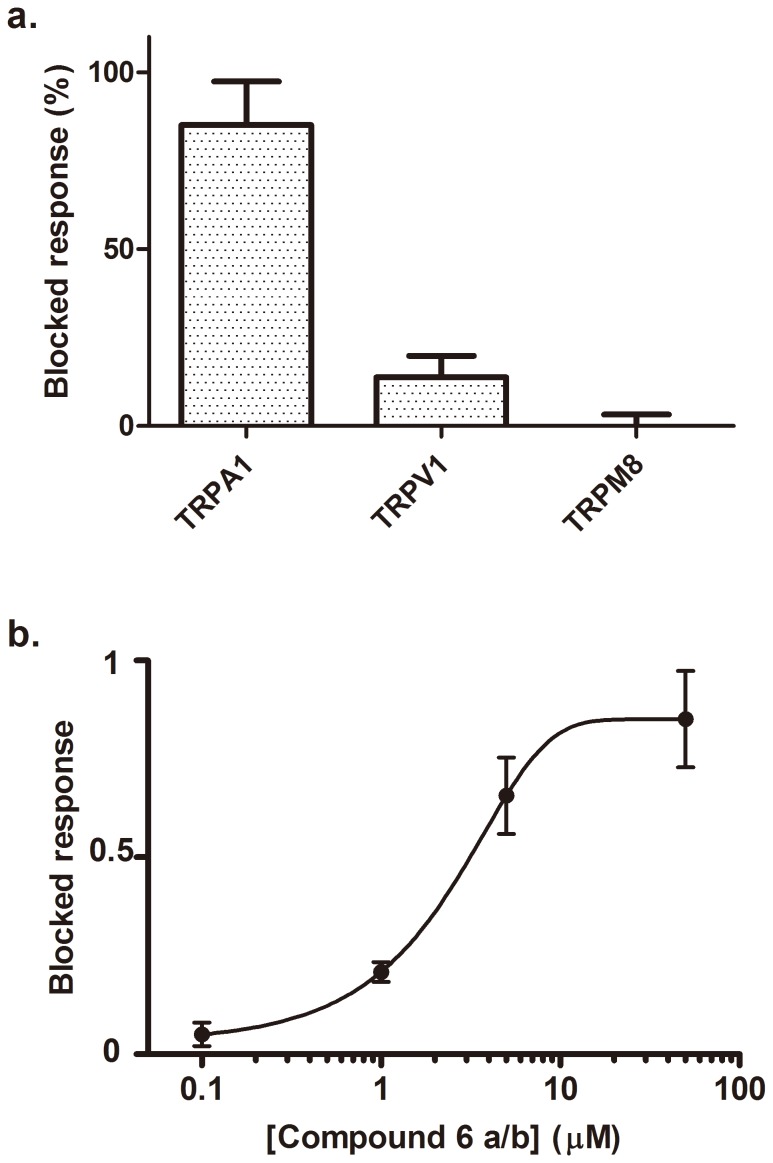
Fluorometric Ca^2+^ assay of thermoTRP channels to evaluate the inhibitory activity of compound 6a/b. a) Activity of compound **6a/b** at 50 µM on TRPA1, TRPV1 and TRPM8. b) Dose-response curve for TRPA1 inhibition by compound **6a/b**. Solid lines depict the best fit to a binding isotherm, with IC_50_  = 2.6±1.1 µM, and n_H_ (hill coefficient)  = 0.21. TRPA1 was activated with 100 µM AITC, TRPV1 was activated by 10 µM capsaicin and TRPM8 was activated with 100 µM menthol. Data are given as mean ± SEM, with n (number of experiments) ≥5.

The channels were stably expressed in eukaryotic cells, and a Ca^2+^ fluorographic assay used to monitor their activity upon instillation of their respective agonists in the absence and presence of compound **6a/b** at 50 µM. As illustrated in Fig. 7a, only the activity of the TRPA1 channels was selectively blocked up to 85% at this concentration of **6a/b**. No significative effect was recorded for the other thermoTRPs. A dose-response curve reveals that compound **6a/b** displayed an IC_50_ of 2.6 µM, indicating that compound **6a/b** is a moderate antagonist of TRPA1 channels (Fig. 7b). The inhibitory activity of compound **6a/b** was further demonstrated electrophysiologically as evidenced by the blockade of the AITC-evoked ionic currents (Figures S1 and S2 in the presence and in the absence of extracellular Ca^2+^). Collectively, these findings indicate that TRPA1 is a molecular target of compound **6a/b**, and could participate in the antinociceptive effect showed in the writing test.

## Conclusions

We have described the synthesis and pharmacological evaluation of new paracetamol analogs derived from an adamantane scaffold. Compounds **5** and **6a/b** represent attractive leads to be developed since they show an improved antinociceptive effect compared to paracetamol. In addition, adamantane derivatives have proved to be very biocompatible, so possible toxic effects due to chronic treatment should not be expected.

The main result of the present communication is that phenyl ring, ubiquitous in medicinal chemistry, in some cases could be replaced by an adamantyl ring without loss but improvement of the biological properties. To the *ortho*, *meta* and *para* positions of a phenyl ring correspond to 1,2, 1,3 and 1,4-substituents on an adamantyl ring. This is in agreement with our findings since the 1,4-derivative, with a similar substitution to that of paracetamol, has shown greater potency than the 1,3-derivative.

Even though more pharmacological research is needed (using TRPA1 agonists for example), compound **6a/b**, an analogue of paracetamol, able to block TRPA1 channel, is an interesting, new, antinociceptive drug.

## Experimental

### Chemistry

All chemicals were purchased from commercial suppliers and used without further purification. TLC: precoated silica-gel 60 254 plates (Merck), detection by UV light (254 nm). Flash-column Chromatography (FC): Kieselgel 60 (230–400 mesh; Merck). Melting points (mp) were determined in open capillaries with a Gallenkamp capillary melting-points apparatus. ^1^H and ^13^C NMR spectra were recorded on Bruker Advance 300 spectrometer operating at 300.13 MHz and 75.47 MHz respectively, in CDCl_3_ or DMSO-*d_6_* as solvents. Chemical shifts are reported in ppm on the *δ* scale. In the case of multiplets, the signals are reported as intervals. Signals were abbreviated as s, singlet; d, doublet; t, triplet; and m, multiplet. Coupling constants are expressed in hertz. Hydrogenation reactions were carried out in a Shaker type hydrogenation apparatus (Parr). Elemental analysis was determined with a LECO Elemental Analyzer CHNS-932. LC-MS analyses were performed using an Alliance 2695 (Waters) with a diode array UV/Vis detector Waters 2996 and interfaced to a Micromass ZQ mass spectrometer. Analyses were performed using reversed phase HPLC silica based columns: column Bridge C18 3.5 mm (2.1×100 mm). Using an injection volume of 3 mL, a flow rate of 0.25 mL/min and gradient elution (2 to 30% over 15 min) of acetonitrile in water. Acetonitrile contains 0.08% v/v formic acid and water contains 0.1% v/v formic acid. Analyses were monitored at 254 nm wavelength.

#### Synthesis of N-3-hydroxyadamantan-1-yl acetamide (5)

To a solution of 3-amino-1-adamantol (0.61 g, 3.7 mmol) in water (7 mL) was added acetic anhydride (0.45 mL) at 0°C. The reaction mixture was kept at 0°C in an ice bath for 1 hour. Then it was taken to room temperature and stirred for 2 hours. When the reaction was completed, the mixture was extracted with CH_2_Cl_2_. The organic layers were dried over MgSO_4_ and the solvent evaporated under reduced pressure yielding a white solid (0.343 g, 45%). M.p. 224.4°C. Elemental analysis calculated for C_12_H_19_NO_2_; C, 68.87; H, 9.15; N, 6.69; found: C, 68.59; H, 9.27; N, 6.86. RMN ^1^H (300 MHz, CDCl_3_) δ 5.25–5.17 (s, 1H), 2.29–2.22 (m, 2H), 2.03–1.95 (m, 2H), 1.95–1.88 (m, 7H), 1.72–1.67 (m, 4H), 1.58–1.53 (m, 2H). ^13^C (75 MHz, CDCl_3_) δ 169.8 (C = O), 69.6 (C-OH), 54.7 (C-NH), 49.5 (CH_2_), 44.5 (2CH_2_), 40.7 (2CH_2_), 35.3 (CH_2_), 31.0 (2CH), 25.0 (CH_3_).

#### Synthesis of 4-((E)-(1-phenylethylidene)amino)adamantan-1-ol (9)

5-Hydroxy-2-adamantanone (**8**) (1.8 g, 10 mmol) and S-α-methylbenzylamine (1.3 g, 10 mmol) were dissolved in absolute ethanol (50 mL) and refluxed for 64 h. The reaction mixture was concentrated obtaining yellow oil as the resulting imine 9 (2.8 g). It was used without further purification in the next step. LC-MS retention time 5.52 min [M+H]^+^  = 270.

#### Synthesis of E and Z-4-aminoadamantan-1-ol (10a) and (10b)

Benzylimine **9** (0.33 g, 1.2 mmol) was dissolved in THF (15 mL) and kept at 0°C in an ice bath for 5 min. Then, Pd-C 10% (0.24 g) was added and the mixture was hydrogenated at atmospheric pressure for 12h. The catalyst was removed by filtration and the solvent evaporated yielding isomers **10a** and **10b** as a white powder (0.13 g, 67%). M.p. 250°C. LC-MS retention time isomer *E* 0.64 min [M+H]^+^  = 168; *Z* isomer 0.95 min [M+H]^+^  = 168. Elemental analysis calculated for C_10_H_17_NO: C, 71.81; H, 10.25; N, 8.37; found: C, 71.60; H, 10.32; N, 8,28. *E*-isomer (**10a**) RMN ^1^H (300 MHz, CDCl_3_) *δ* 3.02–2.98 (m, 1H, CH-NH_2_), 2.11–2.05 (m, 1H, CH(7)), 1.95–1.87 (m, 4H, CH_2_ (6,10), CH (5,3)), 1.80–1.69 (m, 6H, CH_2_ (8,2,9)), 1.41–1.34 (m, 2H, CH_2_ (6,10)). *Z*-isomer (10b) RMN ^1^H (300 MHz, CDCl_3_) *δ* 2.87–2.85 (m, 1H, CH-NH_2_), 2.11–2.05 (m, 1H, CH(7)), 1.99–1.96 (m, 4H, CH (5,3), CH_2_ (2,9)), 1.80–1.69 (m, 2H, CH_2_ (8)), 1.68–1.65 (m, 2H, CH_2_ (6,10)), 1.63–1.58 (m, 2H, CH_2_ (6,10)), 1.53–1.46 (m, 2H, CH_2_ (2,9)). *E*-isomer (10a) ^13^C (75 MHz, CDCl_3_) *δ* 69.6 (C-OH, C1), 54.6 (C-NH, C4), 45.9 (CH_2_, C8), 45.1 (2CH_2_, C2, C9), 37.1 (2CH, C5, C3), 30.3 (2CH_2_, C6, C10), 29.5 (CH, C7). *Z*-isomer (10b) ^13^C (75 MHz, CDCl_3_) *δ* 69.6 (C-OH, C1), 54.0 (C-NH, C4), 45.7 (CH_2_, C8), 38.9 (2CH_2_, C2, C9), 37.9 (2CH, C5, C3), 36.3 (2CH_2_, C6, C10), 29.8 (CH, C7).

#### Synthesis of N-5-hydroxyadamantan-2-yl acetamide (6a) and (6b)

To a solution of isomers **10a** and **10b** (0.50 g, 3 mmol) in water (8 mL) was added acetic anhydride (0.4 mL, 3.5 mmol) at 0°C. The reaction mixture was stirred for 1h at 0°C and then for 2h at room temperature. The mixture was extracted with CH_2_Cl_2_ (10 mL). The organic layers were dried over MgSO_4_ and the solvent evaporated under reduced pressure. The crude was successively washed with ether yielding a mixture of isomers 6a and 6b as a white powder (0.482 g, 77%). M.p. 184°C. LC-MS: LC-MS retention time isomer *E* 7.34 min [M+H]^+^  = 210; isomer Z 7.83 min [M+H]^+^  = 210. Elemental analysis calculated for C_12_H_19_NO_2_: C, 68.87; H, 9.15; N, 6.69; found: C, 68.70; H, 9.35; N, 6.39.

*E*-isomer (**6a**) RMN ^1^H (300 MHz, DMSO-*d*_6_) *δ* 3.75-3.70 (m, 1H, CH-NH), 2.52–2.48 (m, 1H, CH(7)), 1.99–1.91 (m, 2H, CH (5,3)), 1.87–1.81 (m, 5H, CH_2_ (6,10), CH_3_), 1.67–1.60 (m, 2H, CH_2_ (2,9)), 1.60–1.53 (m, 4H, CH_2_ (8,2,9)), 1.28–1.21 (m, 2H, CH_2_ (6,10)). *Z*-isomer (**6b**) RMN ^1^H (300 MHz, DMSO-*d*_6_) *δ* 3.65–3.60 (m, 1H, CH-NH), 2.52–2.48 (m, 1H, CH(7)), 2.00–1.92 (m, 2H, CH (5,3), 1.87–1.81 (m, 5H, CH_2_ (2,9), CH_3_), 1.60–1.53 (m, 6H, CH_2_ (8,6,10)), 1.36–1.29 (m, 2H, CH_2_ (2,9)). *E*-isomer (**6a**) ^13^C (75 MHz, DMSO*-d*_6_). *δ* 169.8 (C = O), 69.6 (C-OH, C1), 52.9 (C-NH, C4), 46.3 (CH_2_, C8), 45.2 (2CH_2_, C2, C9), 34.0 (2CH, C5, C3), 30.5 (2CH_2_, C6, C10), 29.9 (CH, C7) 23.4 (CH_3_, C12). *Z*-isomer (**6b**) ^13^C (75 MHz, DMSO*-d*_6_). *δ* 169.8 (C = O), 69.6 (C-OH, C1), 52.4 (C-NH, C4), 46.0 (CH_2_, C8), 39.8 (2CH_2_, C2, C9), 36.3 (2CH, C5, C3), 34.9 (2CH_2_, C6, C10), 29.8 (CH, C7) 23.3 (CH_3_, C12).

### Pharmacological evaluation

The animals, CD-1 male mice (25–27 g), were redistributed in groups, two of which were used as the control group. The remaining twelve groups were treated with paracetamol 100 and 200 mg/kg (two different groups), compound 5 (100, 200, 500 mg/kg) and compound 6a/b (250, 375, 500, 750 mg/kg). An additional group received morphine 5 mg/kg. All drugs were intraperitoneally (i.p.) administered, 30 minutes before the algogen agent. Control mice were treated with 10 ml/kg of saline solution. Each group included 10–12 animals; those animals showing behavioral alterations were previously discarded. Each animal was used only once. An observer who was unaware of the different treatments carried out the collection of data.

The experimental test used was the acetic acid writhing test. The effect of the i.p. administration of 10 ml/kg of acetic acid (2%) was measured by quantifying the number of writhing responses in the three groups of animals over a 10 minutes period. Responses were measured starting 5 minutes after the algogen agent was administered. The animals used were supplied by the animal house of Universidad Rey Juan Carlos. Room temperature was kept at 22±1°C. A 12 hours light - dark cycle was started at 8:00 a.m., at constant humidity. All animals were given free access to food and water.

Experimental protocols used in this investigation were approved by the Ethical Committee of Rey Juan Carlos University and were conducted in accordance with the guidelines of the International Association for the Study of Pain [49].

#### Inhibition of Isolated Cyclooxygenases COX-1 and COX-2

A COX inhibitor screening assay (kit 700100) was used to determine the activity of isolated ovine COX-1 and human recombinant COX-2 as described by the manufacturer (Cayman Chemical Company, USA).

#### Calcium fluorography

For fluorescence assays, cells expressing TRP channels (TRPV1-SH-SY5Y, TRPM8-HEK and TRPA1-IMR90) were seeded in 96-well plates (Corning Incorporated, Corning, NY) at a cell density of 40,000 cells 2 days before treatment. The day of treatment the medium was replaced with 100 µL of the dye loading solution Fluo-4 NW supplemented with probenecid 2.5 mM. Then the compounds dissolved in DMSO were added at the desired concentrations and the plate(s) were incubated at 37°C in a humidified atmosphere of 5% CO_2_ for 60 minutes.

The fluorescence was measured using instrument settings appropriate for excitation at 485 nm and emission at 535 nm. (POLARstar Omega BMG LAB tech). A baseline recording of 7 cycles was recorded prior to stimulation with the agonist (10 µM capsaicin for TRPV1, 100 µM menthol for TRPM8, and 100 µM AITC for TRPA1). The corresponding antagonist (10 µM Ruthenium Red for TRPV1 and TRPA1, 100 µM AMTB for TRPM8) was added for the blockade. The changes in fluorescence intensity were recorded during 15 cycles more. DMSO, at the higher concentration used in the experiment, was added to the control wells.

The degree of blockage (%) of TRP channel activity was calculated by:
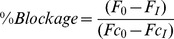



Where:

*F*_0_ is the fluorescence after the addition of agonist in the presence of the compound,

*F_I_* is the fluorescence before the addition of agonist in the presence of the compound,

*Fc*_0_ is the fluorescence after the addition of agonist in the absence of the compound,

*Fc_I_* is the fluorescence before the addition of agonist in the absence of the compound.

The *Z* factor was calculated using the following equation:
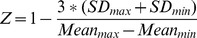



Where:

*Mean*_max_ is the mean of the maximum fluorescence in the presence of agonist,

*Mean*_min_ is the mean of the maximum fluorescence in the presence of agonist and antagonist.

### Computational details

The geometries of compounds **5**, **6a** and **6b** were optimized at the B3LYP/6-31G(d) level and the corresponding frequencies (all 0) proved that they are minima. These geometries were reoptimized at the B3LYP/6-311++G(d,p) level. Absolute shieldings (*σ*, ppm) and SSCC (Hz) were calculated on these geometries using the GIAO approximation for the *σ* values. The corresponding chemical shifts (*δ*, ppm) were obtained using the empirical transformation equations we have established for a large collection of compounds. The used equations transform calculated values in the gas phase to experimental values in solution (usually CDCl_3_ or DMSO-*d_6_*). See supplementary data.

## Supporting Information

Figure S1 **Compound 6a/b strongly inhibits TRPA1- mediated currents in the presence of extracellular Ca^2+^.** **A**) Representative whole-cell voltage clamp recording from hTRPA1-expressing IMR-90. Currents were measured every second during a holding potential of -60 mV. Pre-application of **6a/b** (20 s) was followed by co-application with 100 µM AITC for 60s in presence of 2.0 mM extracellular Ca^2+^. Current traces of different colours denote the different concentrations of compound **6a/b** tested. **B**) Dose response of compound **6a/b** blockade activity. Solid line depicts the fitting to a Michaelis isotherm. The estimated IC_50_ value was 10.6±0.7 µM. Data are given as mean ± sem, with n≥4 cells) per data point.
(TIF)

Figure S2 **Compound 6a/b weakly inhibits TRPA1 currents in the absence of extracellular Ca^2+^**. **A**) Representative whole-cell voltage clamp recording from hTRPA1-expressing IMR-90 cells in the absence of extracellular Ca^2+^. Ionic currents, at positive and negative potentials were measured every 2s during a 350 ms voltage ramp from −60 mV to +60 mV and evoked with 100 µM AITC followed by the addition of 100 µM compound **6a/b** for 20s. AITC was present with drug application and after application to evaluated current reversibility **B**) Current-voltage relationships of TRPA1 in the absence and presence of 100 µM compound **6a/b** in Ca^2+^ free medium. The inferred IC_50_ value was ≥80 µM, as saturation of blockade was not reached in the absence of Ca^2+^. Data were obtained from n≥4 cells.
(TIF)

Table S1 **Absolute chemical shielding for 6a and 6b calculated at GIAO/B3LYP/6-311+G(d,p) level.** The relative values have been obtained using the equation described in ref. 1–2.
(DOC)
