# Supplementary figures and images for: Adamantyl Analogues of Paracetamol as Potent Analgesic Drugs via Inhibition of TRPA1
Source: PLoS One. 2014 Dec 1;9(12):e113841. doi: 10.1371/journal.pone.0113841 (PMC4249970; doi:10.1371/journal.pone.0113841)

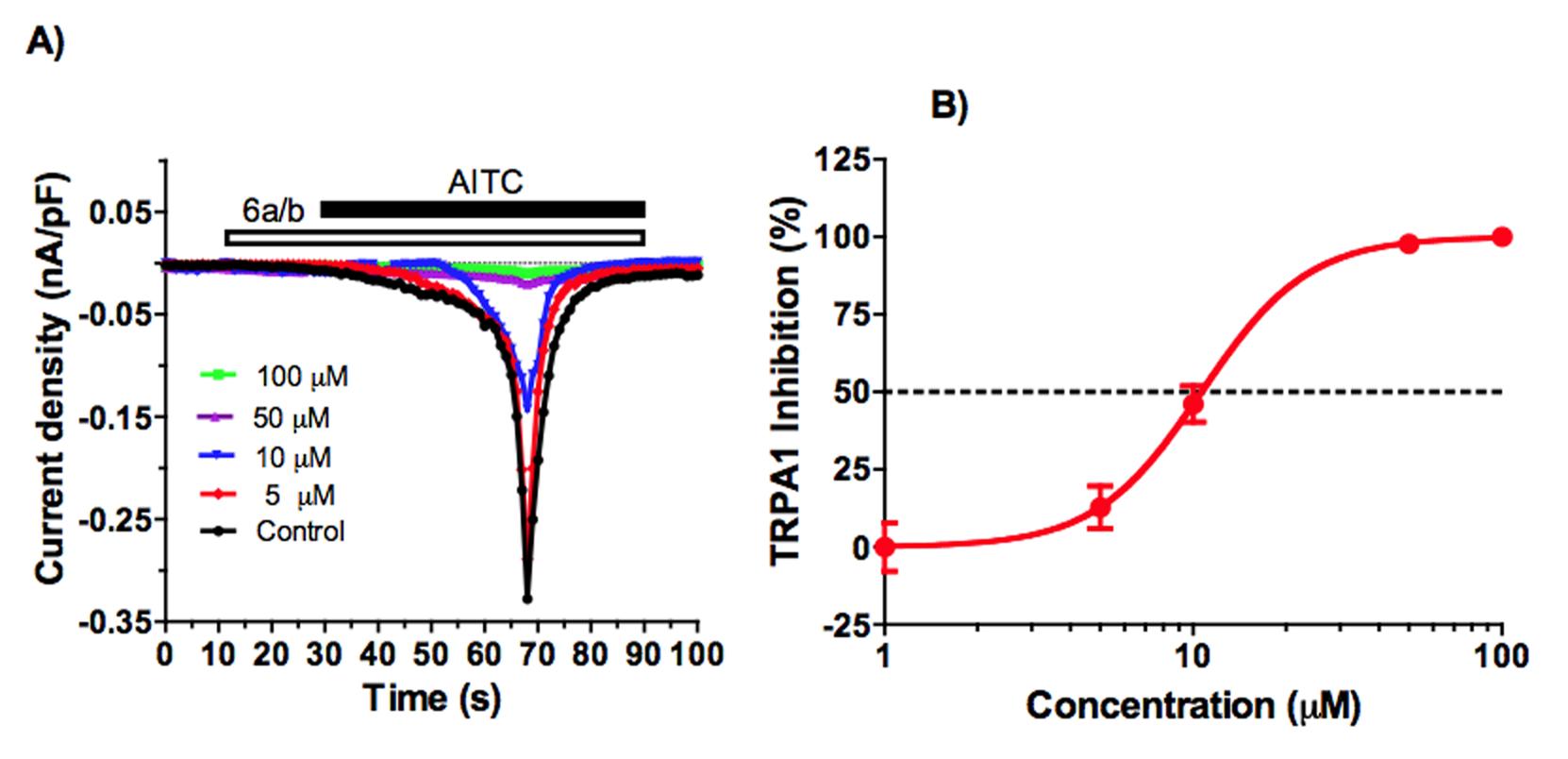

Supplement: Figure S1 — Compound 6a/b strongly inhibits TRPA1- mediated currents in the presence of extracellular Ca2+. A) Representative whole-cell voltage clamp recording from hTRPA1-expressing IMR-90. Currents were measured every second during a holding potential of -60 mV. Pre-application of 6a/b (20 s) was followed by co-application with 100 µM AITC for 60s in presence of 2.0 mM extracellular Ca2+. Current traces of different colours denote the different concentrations of compound 6a/b tested. B) Dose response of compound 6a/b blockade activity. Solid line depicts the fitting to a Michaelis isotherm. The estimated IC50 value was 10.6±0.7 µM. Data are given as mean ± sem, with n≥4 cells) per data point. (TIF) [file pone.0113841.s001.tif]

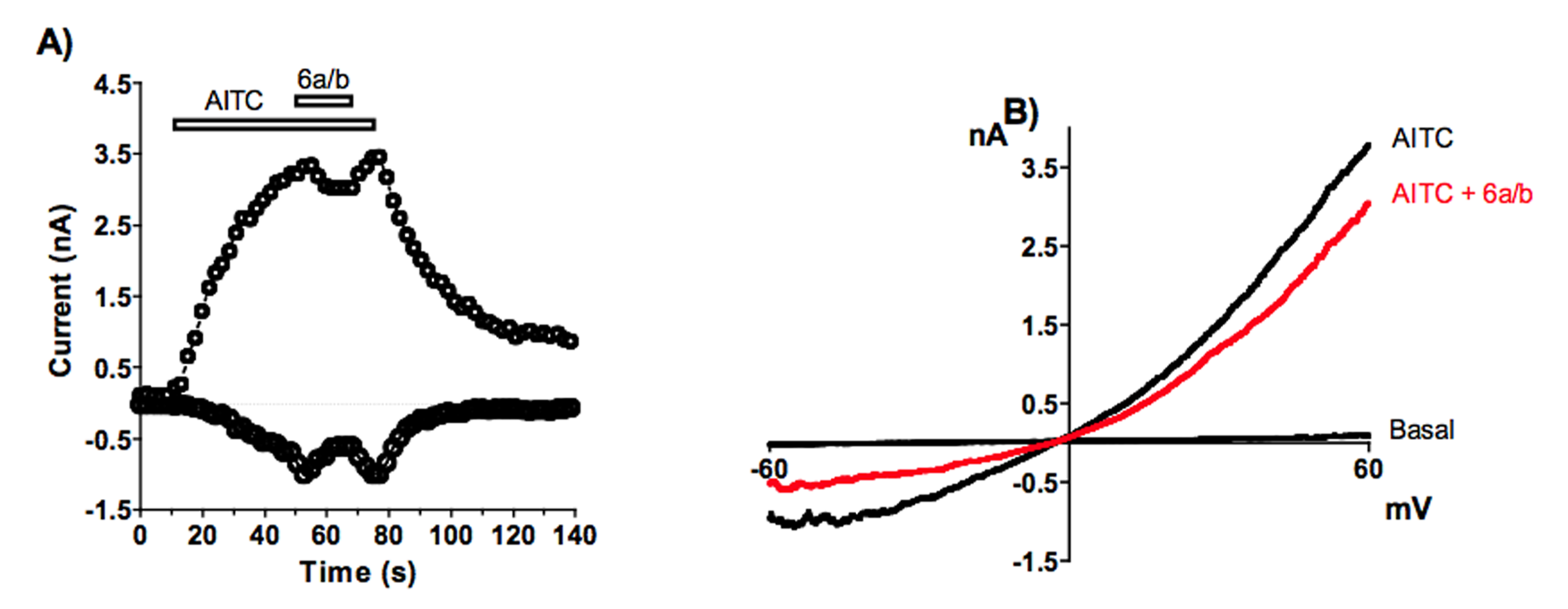

Supplement: Figure S2 — Compound 6a/b weakly inhibits TRPA1 currents in the absence of extracellular Ca2+. A) Representative whole-cell voltage clamp recording from hTRPA1-expressing IMR-90 cells in the absence of extracellular Ca2+. Ionic currents, at positive and negative potentials were measured every 2s during a 350 ms voltage ramp from −60 mV to +60 mV and evoked with 100 µM AITC followed by the addition of 100 µM compound 6a/b for 20s. AITC was present with drug application and after application to evaluated current reversibility B) Current-voltage relationships of TRPA1 in the absence and presence of 100 µM compound 6a/b in Ca2+ free medium. The inferred IC50 value was ≥80 µM, as saturation of blockade was not reached in the absence of Ca2+. Data were obtained from n≥4 cells. (TIF) [file pone.0113841.s002.tif]
