# Supplementary material for: Adamantyl Analogues of Paracetamol as Potent Analgesic Drugs via Inhibition of TRPA1
Source: PLoS One. 2014 Dec 1;9(12):e113841. doi: 10.1371/journal.pone.0113841 (PMC4249970; doi:10.1371/journal.pone.0113841)
Supplement: Table S1 — Absolute chemical shielding for 6a and 6b calculated at GIAO/B3LYP/6-311+G(d,p) level. The relative values have been obtained using the equation described in ref. 1–2. (DOC) [file pone.0113841.s003.doc]

**Table S1**. Absolute chemical shielding for **6a** and **6b** calculated at GIAO/B3LYP/6-311+G(d,p) level. The relative values have been obtained using the equation described in ref. 1-2.

**6a**

Atom Abs. Rel.

1C 132.43 48.17

2H 30.12 1.78

3H 30.38 1.53

4C 142.69 38.29

5H 30.18 1.72

6C 110.63 69.17

7C 136.94 43.83

8H 30.17 1.74

9H 30.16 1.75

10C 147.68 33.49

11H 30.44 1.47

12H 30.36 1.55

13C 147.56 33.60

14H 30.00 1.90

15C 148.42 32.77

16H 30.50 1.42

17H 30.23 1.68

18C 126.52 53.86

19H 27.88 3.95

20C 133.80 46.85

21H 30.36 1.55

22H 30.16 1.75

23C 143.24 37.76

24H 29.94 1.96

25O 226.26

26H 32.01 -0.05

27N 112.34 -258.27

28H 26.80 5.00

29C 12.27 163.89

30O -73.88

31C 159.69 21.92

32H 30.63 1.29

33H 29.99 1.91

34H 29.97 1.93

**6b**

1C 136.92 43.85

2H 30.20 1.70

3H 30.74 1.18

4C 141.96 38.99

5H 30.12 1.78

6C 110.49 69.30

7C 137.00 43.76

8H 30.20 1.70

9H 30.21 1.70

10C 142.29 38.67

11H 30.20 1.71

12H 30.22 1.68

13C 147.76 33.41

14H 30.02 1.88

15C 143.83 37.19

16H 30.27 1.64

17H 30.18 1.73

18C 126.81 53.58

19H 28.01 3.83

20C 137.85 42.95

21H 30.49 1.42

22H 30.34 1.57

23C 141.80 39.15

24H 29.81 2.08

25O 221.18

26H 32.06 -0.10

27N 110.17 -256.22

28H 26.88 4.93

29C 12.41 163.75

30O -75.04

31C 159.76 21.86

32H 30.65 1.27

33H 29.97 1.93

34H 29.98 1.92

References:

1. Silva A M S, Sousa R M S, Jimeno M L, Blanco F, Alkorta I, Elguero J (2008) Experimental measurements and theoretical calculations of the chemical shifts and coupling constants of three azines (benzalazine, acetophenoneazine and cinnamaldazine), Magn Reson Chem 46: 859-864. doi: 10.1002/mrc.2272

2. Blanco F, Alkorta I, Elguero J (2007) Statistical analysis of 13C and 15N NMR chemical shifts from GIAO/B3LYP/6-311++G** calculated absolute shieldings, Magn Reson Chem 45:797:800. doi: 10.1002/mrc.2053
